# Dynamin-related proteins in plant post-Golgi traffic

**DOI:** 10.3389/fpls.2014.00408

**Published:** 2014-09-04

**Authors:** Masaru Fujimoto, Nobuhiro Tsutsumi

**Affiliations:** Laboratory of Plant Molecular Genetics, Graduate School of Agricultural and Life Sciences, The University of TokyoTokyo, Japan

**Keywords:** dynamin, dynamin-related protein, plant, post-Golgi traffic, cytokinesis

## Abstract

Membrane traffic between two organelles begins with the formation of transport vesicles from the donor organelle. Dynamin-related proteins (DRPs), which are large multidomain GTPases, play crucial roles in vesicle formation in post-Golgi traffic. Numerous *in vivo* and *in vitro* studies indicate that animal dynamins, which are members of DRP family, assemble into ring- or helix-shaped structures at the neck of a bud site on the donor membrane, where they constrict and sever the neck membrane in a GTP hydrolysis-dependent manner. While much is known about DRP-mediated trafficking in animal cells, little is known about it in plant cells. So far, two structurally distinct subfamilies of plant DRPs (DRP1 and DRP2) have been found to participate in various pathways of post-Golgi traffic. This review summarizes the structural and functional differences between these two DRP subfamilies, focusing on their molecular, cellular and developmental properties. We also discuss the molecular networks underlying the functional machinery centering on these two DRP subfamilies. Furthermore, we hope that this review will provide direction for future studies on the mechanisms of vesicle formation that are not only unique to plants but also common to eukaryotes.

## INTRODUCTION

Eukaryotic cells are distinguished by the presence of internal membrane-bound organelles, including mitochondria, peroxisomes, plastids, and other single membrane-bound organelles [e.g., the endoplasmic reticulum (ER), Golgi apparatus, *trans*-Golgi network (TGN), plasma membrane (PM), and a series of endosomal compartments]. These single membrane-bound organelles are connected with each other through a membrane trafficking system mediated by vesicular and/or tubular membranous transport carriers. Membrane traffic consists of four sequential processes: the formation of cargo-bearing vesicles or tubules from the donor membrane, targeted delivery of transport carriers, tethering of carriers to target membranes and membrane fusion (Bonifacino and Glick, 2004). Especially, in post-Golgi traffic, the formation of transport vesicles from the donor organelle is accomplished by snipping the neck of the invaginated membrane by dynamin-related proteins (DRPs; McMahon and Mills, 2004; Praefcke and McMahon, 2004). DRPs are large multidomain GTPase that regulate membrane fission, fusion, and tabulation during diverse cellular activities such as endocytosis, cytokinesis, vacuolar sorting, fission and fusion of mitochondria, biogenesis of peroxisomes, and the maintenance of ER morphology (Heymann and Hinshaw, 2009; McNew et al., 2013). This review summarizes recent advances in understanding how DRPs are involved in post-Golgi traffic, focusing on the unique aspects of the plant system.

## DYNAMIN, MEMBRANE-SCISSION CATALYST IN ANIMALS

Presently, the best characterized DRPs are animal dynamins that act in post-Golgi clathrin-mediated traffic (Praefcke and McMahon, 2004; Ferguson and De Camilli, 2012). During clathrin-coated vesicle (CCV) formation, dynamin is thought to assemble into helical or ring shaped-structures at the neck of clathrin-coated membrane buds (Takei et al., 1995), and constrict to sever the bud neck membrane in a GTP hydrolysis-dependent manner (Sweitzer and Hinshaw, 1998; Macia et al., 2006). Much progress has been made in dynamin function using *in vitro* analyses (Chappie and Dyda, 2013). Purified dynamin assembles into a ring and a spiral-shaped structure with 40 ∼ 50 nm outer diameter (Hinshaw, 2000; Faelber et al., 2011; Ford et al., 2011). The intramolecular conformational change of dynamin with the activation of its GTPase reduces the dynamin helix diameter (Stowell et al., 1999; Danino et al., 2004). However, attempts to directly observe dynamin-spiral formation and constriction in living cells have been unsuccessful thus far.

**FIGURE 1:**
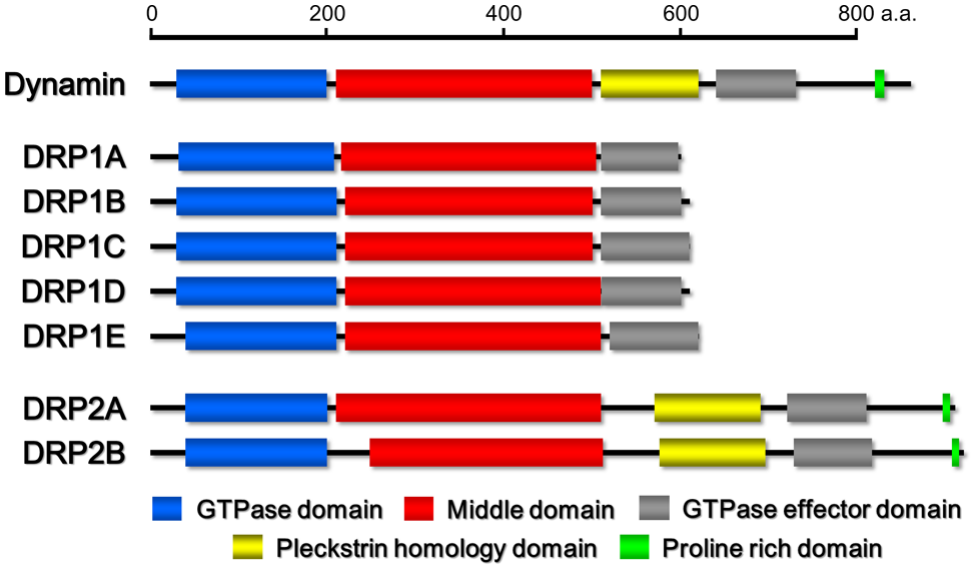
**Schematic drawings of the domain organizations of *Homo sapiens* dynamin and *Arabidopsis thaliana* DRP1 and DRP2 family members.** The domains were identified by the pfam program (http://pfam.sanger.ac.uk/).

On the basis of its sequence, dynamin harbors five distinct domains (**Figure 1**): an amino terminal GTPase domain, whose activation causes the intramolecular conformational change of dynamin (Ford et al., 2011), a middle domain, which mediates the intermolecular interaction between dynamins during self-assembly (Ramachandran et al., 2007), GTPase effector domain (GED), which stimulates the GTPase activity (Sever et al., 1999), a pleckstrin homology domain (PH domain) that may participate in the generation of membrane curvature and the breakdown of the lipid bilayer through the binding of acidic phospholipids and phosphatidyl inositol-4,5-bisphosphate (PI4,5P_2_) on the donor membrane (Ferguson et al., 1994; Zheng et al., 1996; Schmid and Frolov, 2011), and a carboxy-terminal proline-rich domain (PRD) harboring an array of PXXP amino acid motifs, which interact with many Src homology 3 (SH3) domain-containing proteins to localize dynamin at vesicle formation sites (Shpetner et al., 1996; Grabs et al., 1997). The former three domains (GTPase domain, middle domain and GED) are conserved among almost all DRP proteins. However, DRPs with a domain configuration similar to that of dynamin, which also harbor additional domains, have been found only in Metazoans and Embryophyta (Chanez et al., 2006; Miyagishima et al., 2008; Heymann and Hinshaw, 2009).

X-ray crystallographic studies have provided insights into the spatial structure and disposition of each domain within the dynamin spiral polymer (Chappie et al., 2011; Faelber et al., 2011; Ford et al., 2011). The GTPase domain is placed at the outside of the spiral and interacts with the GTPase domain of dynamin in the adjacent turns of dynamin spiral. The PRD may protrude outward from the dynamin spiral structure (Ferguson and De Camilli, 2012). In contrast, the PH domain sits at the inside of the spiral, which is known as the “foot” region. This location is consistent with the expected function of the PH domain. The middle domain and the *N*-terminal region of the GED interact to form an intramolecular helical “stalk” region which is located between the GTPase and PH domains and is responsible for the dimerization of dynamin. This dimer in which the two GTPase domains are oriented in opposite directions is the basic unit for dynamin polymerization. The *C*-terminal region of the GED associates with the *N*- and *C*-terminal helix of the GTPase domain to assemble into a helix bundle, referred to as “neck” region, which modulates the GTPase activity of dynamin *in vitro* (Chappie et al., 2009).

## DYNAMIN-RELATED PROTEINS IN LAND PLANTS

**FIGURE 2:**
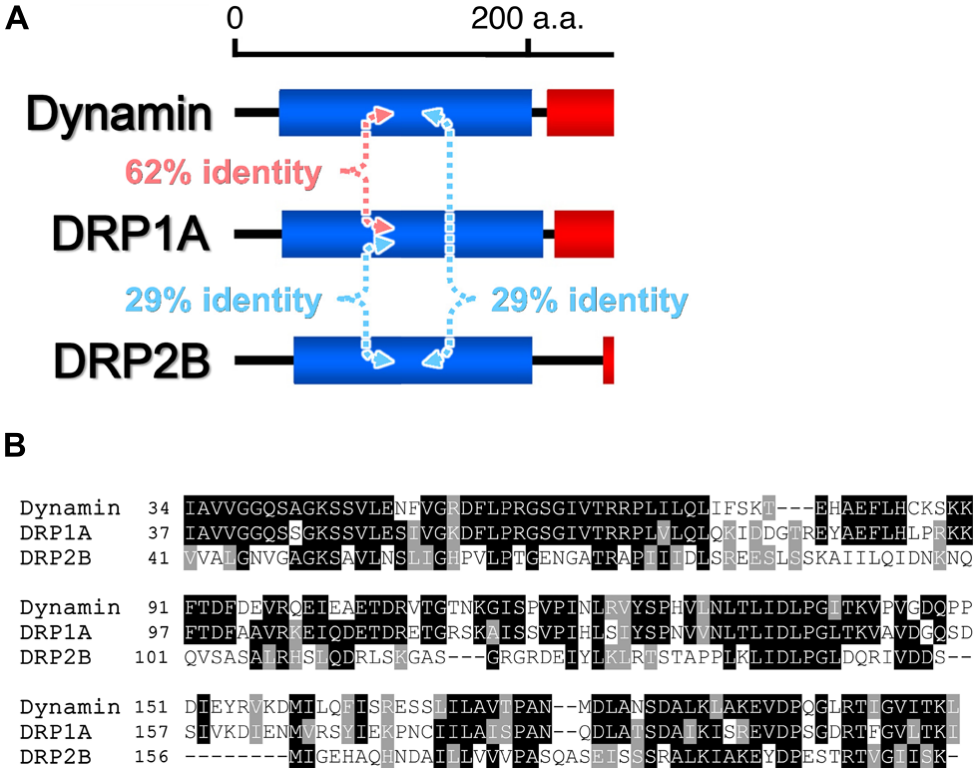
**Comparisons of the amino acid sequences of the GTPase domains of *H. sapiens* dynamin and *Arabidopsis thaliana* DRP1A and DRP2B. (A)** Pairwise amino acid sequence identities. **(B)** Clustal alignment of the sequences. Identical and similar amino acid residues are outlined in black and gray, respectively.

Based on phylogenetic analyses and the domain-structure predictions, the genomes of most land plants contain six types of DRPs: DRP1–DRP4, DRP5A, and DRP5B (Hong et al., 2003a; Miyagishima et al., 2008). Over the last two decades, much progress has been made in elucidating the functions of most of the plant DRPs (the function of DRP4 is still unclear). DRP3 is conserved in a wide range of Eukaryota and is involved in mitochondrial fission (Fujimoto et al., 2009). DRP5B is conserved in a wide range of Archaeplastida and is involved in plastid division (Gao et al., 2003; Miyagishima et al., 2003). Both DRP3 and DRP5B are also involved in fission and biogenesis of peroxisomes (Mano et al., 2004; Zhang and Hu, 2010). DRP5A, which closely resembles DRP5B in sequence and domain structure, is distributed in Viridiplantae, Amoebozoa, and Heterolobosea, in which it appears to participate in cytokinesis including cell plate formation (Miyagishima et al., 2008), although molecular functions of DRP5A is unclear. Thus, the functions of DRP5A and DRP5B appear to be different despite their close structural similarity. DRP1 and DRP2 are found in Viridiplantae and Embryophyta, respectively (Lopez-Bautista et al., 2003; Miyagishima et al., 2008). The phylogenetic distribution of DRP1 is wider than that of DRP2 (Hong et al., 2003a). Both proteins function in several types of post-Golgi traffic pathways: clathrin-mediated endocytosis (CME; Konopka et al., 2008; Fujimoto et al., 2010; Taylor, 2011) and cell plate formation (Hong et al., 2003b; Kang et al., 2003a; Fujimoto et al., 2008). DRP2 also participates in vacuolar trafficking (Jin et al., 2001). Interestingly, the overall domain organization of Embryophyta-specific DRP2s is similar to that of animal dynamins (Hong et al., 2003a; **Figure 1**). DRP1 lacks a PH domain and PRD (Hong et al., 2003a; **Figure 1**). Despite the similarity in the domain organizations of DRP2 and animal dynamin, the GTPase domain of animal dynamin is more similar to the GTPase domain of DRP1 than to that of DRP2 (e.g., animal dynamin has 62% identity to *Arabidopsis thaliana* DRP1A and only 29% identity to *A. thaliana* DRP2B; **Figure 2**).

## *IN PLANTA* FUNCTIONS OF DRP1 AND DRP2

**FIGURE 3:**
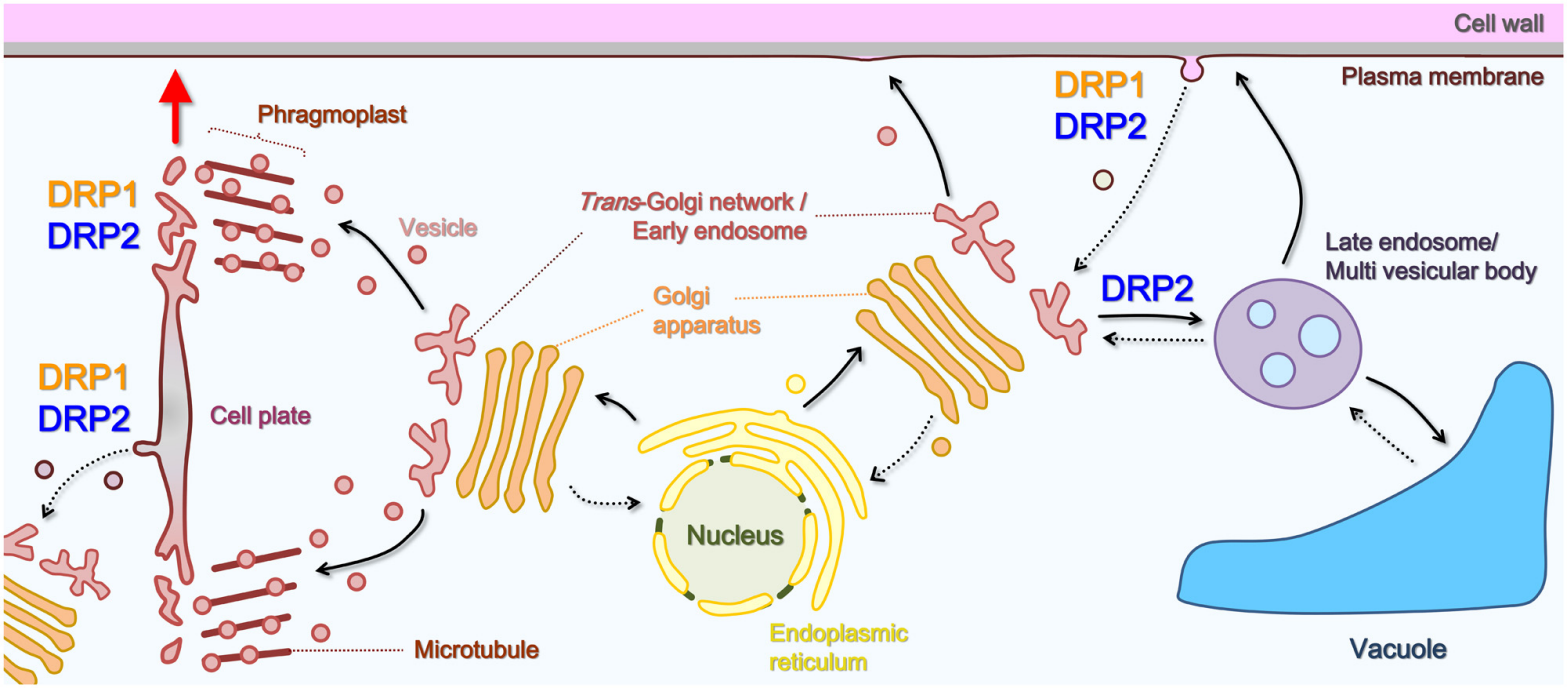
**Schematic illustration of the roles of DRP1 and DRP2 in post-Golgi trafficking pathways.** Solid black arrows, secretory or vacuolar pathways; dotted black arrows, endocytic or retrograde pathway; red arrow, direction of cell plate expansion.

Viridiplantae-specific DRP1 was originally identified as a dynamin-related GTPase, called phragmoplastin, that was associated with the formation of the cell plate in *Glycine max* (Gu and Verma, 1996). The gene was first sequenced in *A. thaliana* (Dombrowski and Raikhel, 1995). *A. thaliana* has five DRP1s (DRP1A–DRP1E; Kang et al., 2001; Hong et al., 2003a; **Figure 1**) that share a high degree (63–82%) of amino acid sequence identity. Experiments with DRP1 mutants showed that the five DRP1s have distinct *in planta* roles, mainly as a result of differences in their spatio-temporal expression patterns and levels (Bednarek and Backues, 2010). Loss-of-function mutants of these members exhibit pleiotropic developmental and cellular phenotypes: *drp1a* and/or *drp1e* null mutants have defects of cell plate formation in root and arrest of embryo development, which suggests that DRP1A and DRP1E participate in cytokinetic membrane trafficking or remodeling (Otegui et al., 2001; Kang et al., 2003a; **Figure 3**). *drp1a* null mutant also takes up less of an endocytic marker (FM4-64; Collings et al., 2008), does not restrict a cytokinesis-related Qa-SNARE protein (KNOLLE) to the division plane (Boutte et al., 2010) and has an altered cell wall composition (Collings et al., 2008). In addition, *drp1c* mutant pollen grains and in *drp1a* mutant stigmatic papillae also have aberrant PM invaginations and defects in cell expansion (Kang et al., 2003a,b). These mutant phenotypes suggest that DRP1 is involved in endocytic and/or recycling trafficking of PM lipids and proteins (**Figure 3**).

On the other hand, genes encoding DRP2 (*DRP2A* and *DRP2B*) were initially identified in the *A. thaliana* genome (Mikami et al., 2000; **Figure 1**). DRP2s appear to be unique to embryophytes. DRP2A and DRP2B have high sequence identity (92%). They are expressed ubiquitously but most strongly around root apical meristems and vascular bundles (our unpublished results). No loss-of-function phenotypes have been detected in *drp2a* or *drp2b* mutants under laboratory conditions, which suggests that the two proteins have functional redundancy (Backues et al., 2010; Taylor, 2011). However, the double mutation of *drp2a* and *drp2b* caused defects in male and female gametogenesis with aberrant Golgi cisterna and alteration of cell wall composition and structure (Backues et al., 2010), which was also detected in a loss-of-function mutant of one of three members of the DRP2 subfamily in *Oryza sativa* (Hirano et al., 2010; Xiong et al., 2010). Moreover, the transient and inducible expression of mutated DRP2A and/or DRP2B, which harbor an amino acid substitution resulting in the loss of GTPase activity, have dominant-negative effects on the TGN-to-vacuole trafficking of some soluble and membrane cargoes (Jin et al., 2001) and on the uptake of FM4-64 from the PM (Taylor, 2011). These loss-of-function phenotypes imply that DRP2 participates in multiple pathways of post-Golgi trafficking (**Figure 3**).

## SUBCELLULAR LOCALIZATION AND DYNAMICS OF DRP1 AND DRP2

Observations with confocal microscopy clearly showed that DRP1 is localized to the cell plate, in agreement with the finding that it is required for cell plate formation (Gu and Verma, 1996, 1997; Kang et al., 2003a,b; Collings et al., 2008). During cytokinesis, DRP1 mainly localizes to the newly synthesized edge region of the forming cell plate (Hong et al., 2003b; Fujimoto et al., 2008). Otegui et al. (2001) examined the localization of antibody-labeled *A. thaliana* DRP1A in dividing endosperm cells with high resolution electron tomography. They detected antibody-labeled spiral-shaped structures constricting the membranous tubular networks in the region where the cells were dividing. Interestingly, a large amount of CCVs and buds were also observed just inside the leading edge of the cell plate, which may be involved in removing and recycling excess membrane materials (Samuels et al., 1995; Otegui et al., 2001; Segui-Simarro et al., 2004). These findings suggest DRP1 is involved in membrane remodeling and vesicle formation at the forming cell plate (**Figure 3**).

Total internal reflection fluorescence microscopy (TIRFM) is an optical technique for observing fluorescence in the cellular surface layer very close to the cover glass (100–400 nm from the cover glass; Toomre and Manstein, 2001). In animal and yeast cells, TIRFM has provided live imaging of endocytosis-related molecules that act near the PM, such as dynamin and clathrin (Merrifield et al., 2002; Rappoport and Simon, 2003). In plant cells surrounded by a cell wall, the fine localizations and dynamics of DRP1 and DRP2 was also visualized using a TIRFM-related technique called variable angle epifluorescence microscopy (VAEM) or variable incidence angle fluorescence microscopy (VIAFM). In VIAFM images of *A. thaliana* root epidermal cells and cultured cells, fluorescently labeled DRP1 forms discrete and mobile foci whose diameters are 200–500 nm and partially co-localizes with fluorescent fusions of the clathrin light chain (CLC; Fujimoto et al., 2007; Konopka et al., 2008; Konopka and Bednarek, 2008; Fujimoto et al., 2010), which is similar to those of animal dynamin (Merrifield et al., 2002). The localizations and dynamics of DRP1 foci at the PM are affected by several compounds. These include some endocytic inhibitors (Konopka et al., 2008; Fujimoto et al., 2010), Tyrphostin A23, which perturbs the interaction between the cargo and the AP-2 clathrin adaptor complex (Banbury et al., 2003) and fenpropimorph, which inhibits the biosynthesis of sterols that mediate the assembly of the endocytic machinery and its cargo at the PM (He et al., 2003; Pichler and Riezman, 2004; Schrick et al., 2004). These results suggest that DRP1 is involved in the formation of endocytic vesicles (**Figure 3**).

**FIGURE 4:**
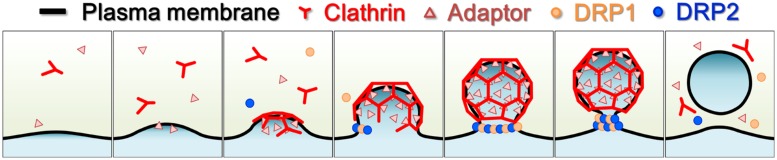
**Schematic illustration of endocytic clathrin-coated vesicle formation in land plants.** Light yellow, cytosol; black line, plasma membrane; red, clathrin and clathrin coat; pink triangles, clathrin adaptors; orange dots, DRP1; blue dots, DRP2.

Confocal and electron microscopy studies of fluorescently labeled DRP2 have shown that DRP2 has a broader localization than DRP1. Although DRP2, like DRP1, localizes to the cell plate and the PM, it also localizes to other post-Golgi organelles such as Golgi/TGN/endosomes (Jin et al., 2001; Lam et al., 2002; Fujimoto et al., 2008; Taylor, 2011). Moreover, VIAFM observations using *A. thaliana* root epidermal and cultured cells revealed that fluorescently labeled DRP2 also forms into discrete foci that partially co-localize with fluorescently labeled CLCs (Fujimoto et al., 2010). These observations suggest that DRP2 participates in multiple post-Golgi trafficking pathways including endocytosis (**Figure 3**). Interestingly, in a time-course confocal laser scanning microscopy (CLSM) analysis, fluorescently labeled DRP2 and DRP1 closely co-localized at the newly formed edge and the central matured region of the cell plate throughout cytokinesis (Fujimoto et al., 2008). Moreover, a VIAFM analysis showed that fluorescently labeled DRP2 and DRP1 largely co-localize with each other in discrete foci and assemble/disassemble together at the PM (Fujimoto et al., 2010). These spatiotemporal relationships between DRP1 and DRP2 raise the hypothesis that these two structurally different DRPs act coordinately in membrane remodeling and CCV formation during cytokinesis and endocytosis (**Figure 4**).

## MOLECULAR NETWORK UNDERLYING THE FUNCTIONAL MACHINERY FOR DRP1 AND DRP2

As described above, dynamin *in vitro* polymerizes into a ring- and a spiral-shaped structure. However, *in vivo*, dynamin has been reported to form a functional complex with various proteins for membrane curvature sensing/generating, cargo concentrating/selecting and so on (McMahon and Boucrot, 2011). Dynamin also associates with the membrane lipids to localize to the vesicle formation site where it snips the budding membrane at its neck (Schmid and Frolov, 2011).

Genomic and phylogenetic analyses indicate that plants rarely have obvious orthologs of dynamin-interacting proteins (Holstein, 2002; Chen et al., 2011; De Craene et al., 2012; Gadeyne et al., 2014). However, these studies also show that many plant proteins contain domains for membrane curvature sensing/generation, membrane lipid association and cargo selection. In animals, such domains are found in protein–protein interaction networks centering on dynamin (McMahon and Boucrot, 2011). For example, an *A. thaliana* protein (VAN3) contains a Bin/amphiphysin/Rvs (BAR) domain, which recognizes and/or induces membrane curvature (Suetsugu et al., 2010). VAN3 has been shown to interact with DRP1 via yeast-two hybrid (Y2H) and co-immunoprecipitation (Co-IP; Koizumi et al., 2005; Sawa et al., 2005). VAN3, which localizes to TGN and PM (Naramoto et al., 2009, 2010) also has two other domains. One is an ADP-ribosylation factor-GTPase activating protein (ARF-GAP) domain that regulates vesicle formation in post-Golgi traffic (Spang et al., 2010). The other is a PH domain, which associates with a membrane phospholipid, phosphatidylinositol 4-phosphate (PI4P) that accumulates at TGN and PM (Koizumi et al., 2005; Simon et al., 2014). Fluorescently labeled DRP1 and VAN3 co-localize at the TGN in *A. thaliana* protoplasts, and a *drp1a* mutant has a vascularization defect that is also observed in a *van3* mutant (Koizumi et al., 2005; Sawa et al., 2005). These findings imply that DRP1 also participates in multiple trafficking pathways around TGN. Moreover, DRP1 has been shown to interact with a putative trafficking cargo protein, the auxin eﬄux carrier PIN1, in *A. thaliana* via Co-IP, bimolecular fluorescence complementation (BiFC) and fluorescence resonance energy transfer (FRET; Mravec et al., 2011). DRP1 also binds to two other cargo proteins, cell plate-specific callose synthase and UDP-glucose transferase in *G. max* via Co-IP and Y2H (Hong et al., 2001a,b). The interactions between DRP1 and trafficking cargos raise the possibility that DRPs, which are unique to plants, function in vesicle formation, such as in selecting or concentrating the vesicle’s cargo or in sensing the progression of vesicle formation.

Some proteins have been identified as interacting partners of DRP2 via Y2H screening with the *C*-terminal half of *A. thaliana* DRP2A. One of them is AtSH3P3, which was shown to bind to the PRD of DRP2A (Lam et al., 2002). AtSH3P3 has a SH3 domain that is known to be a PRD-binding motif (Li, 2005; Kaneko et al., 2008). SH3 domains are also found in animal amphiphysins, which participate in CME through their BAR-domain (Daumke et al., 2014). The *A. thaliana* genome encodes three SH3-domain containing proteins. Although these proteins lack a BAR-domain, they are thought to be involved in clathrin-mediated trafficking (Lam et al., 2001). DRP2 has another proline rich region in the GED, which interacts with g-adaptin (Lam et al., 2002). g-Adaptin is a subunit of the AP1-clathrin adaptor complex that participates in late secretory and vacuolar traffic around the TGN (Park et al., 2013; Teh et al., 2013; Wang et al., 2013). The *C*-terminal half of *A. thaliana* DRP2A also interacts with yeast Sec13 homolog, AtSeh1, which specifically binds to the PH domain of DRP2, apparently to regulate its phosphoinositide interaction (Lee et al., 2006). The TPLATE complex, which is also referred to as TSET, is an endocytic clathrin adaptor that is conserved across a wide range of eukaryotes (Hirst et al., 2014). Co-IP assays have shown that some subunits of this complex interact with both DRP1 and DRP2 in *A. thaliana* (Gadeyne et al., 2014). This strengthens the possibility that DRP2 participates in CME in a manner similar to that of DRP1. Additional protein–protein interaction screening will be needed to elucidate the functions of DRP1 and DRP2.

Most DRP family proteins undergo homo-polymerization (Praefcke and McMahon, 2004). Y2H assays have shown that DRP1 and DRP2 interact, which indicates that structurally distinct DRPs can form hetero-polymers (Hong et al., 2003b; Fujimoto et al., 2010). As mentioned above, DRP3 and DRP5B are involved in the division of mitochondria and plastids, respectively. A Co-IP assay in *A. thaliana* plants raised the possibility that DRP3 and DRP5B form a heteromeric complex that is involved in the fission of peroxisomes (Zhang and Hu, 2010). However, the hetero-polymerization of DRPs belonging to different subfamilies has not been reported in other eukaryotic lineages. Further studies using Co-IP, BiFC, or FRET are needed to verify the heteropolymerization of DRP1 and DRP2.

Phosphoinositide within the cytosolic side leaflet of each post-Golgi organelle membrane participates in the assembly and disassembly of membrane traffic machinery (Krauss and Haucke, 2007; Mayinger, 2012). PI4, 5P_2_, which is the main binding target of the PH domain of dynamin (Ferguson et al., 1994; Zheng et al., 1996; Schmid and Frolov, 2011), functions in the recruitment and assembly of dynamin to the site of vesicle formation (Antonescu et al., 2011; Ferguson and De Camilli, 2012). Although DRP1 lacks a PH domain and other domains known to associated with phosphoinositides, liposome-binding *in vitro* studies suggest that *A. thaliana* DRP1A interacts with three kinds of phosphoinositides (Backues and Bednarek, 2010). One is phosphatidylinositol 3-phosphate (PI3P), which is localized in endosomes/prevacuolar compartments (PVCs) and the vacuole (Vermeer et al., 2006; Simon et al., 2014). Another is PI4P, which accumulates mainly in the TGN, PM and the newly synthesized part of the cell plate (Vermeer et al., 2009; Simon et al., 2014). The third is PI5P, whose subcellular localization is unclear (Krauss and Haucke, 2007). In contrast to DRP1, *A. thaliana* DRP2 has a PH domain. Protein–lipid overlay assays and liposome sedimentation assays suggest that the PH domain interacts with PI3P (Lee et al., 2002; Backues and Bednarek, 2010), PI4P (Lam et al., 2002; Lee et al., 2002; Backues and Bednarek, 2010), and PI4, 5P_2_ (Lam et al., 2002) which is localized specifically to the PM (Simon et al., 2014). These multiple phosphoinositide interactions of DRP2 are consistent with its wide distribution in post-Golgi organelles. However, the effects of multiple phosphoinositide interactions of DRP1, which has a narrower distribution than DRP2, on its subcellular localization remain unclear. Further studies are needed to understand the changes in the subcellular localizations of these two DRPs and phosphoinositide during developmental and physiological acclimation.

## PERSPECTIVES

The above findings show that DRP1 and DRP2 share several properties, including subcellular co-localization, intermolecular interaction, and membrane phospholipid binding similarity. This raises the possibility that DRP1 and DRP2 act together in a heteromeric complex to form the vesicles used in post-Golgi traffic (**Figure 4**). They also appear to work together in cell plate formation, in contrast to metazoan dynamins which perform this function by themselves. However, this hetero-polymerization of structurally distinct DRPs has so far been observed only in embryophytes. This raises three major questions about the embryophyte-unique hetero-DRP complex. First, how does this heteromeric complex bend or sever the organelle membrane? To answer this question, the effects of DRP1 and DRP2 and their GTPase-defective mutants on the morphology of the lipid bilayer need to be compared both *in vitro* and *in vivo*. The tertiary structures of DRP1 and DRP2 also need to be determined. Second, if a heteromeric complex of DRP1 and DRP2 has roles in both membrane remodeling and severing, how does it switch from one role to the other? To answer this question, studies are needed to determine the molecular ratios of DRP1 and DRP2 in the heteromeric complex at the leading edge and in the mature region of the cell plate and in other post-Golgi organelles. DRP1 and DRP2 have different kinetic velocities of GTPase activity (Lam et al., 2002; Sawa et al., 2005), which may be due to a structural difference in the GTPase domain. Third, what is the reason for the evolutionary development of a heteromeric complex in embryophytes? Three studies are needed to answer this question: determine the phenotype caused by the partial-loss of DRP2 function, identify other proteins that interact with DRP2 and clarify how vesicles are formed in Chlorophyta, which do not have DRP2.

In addition to questions about the embryophyte-unique DRP complex, two fundamental questions about DRP assembly remain to be solved. First, does the DRP that is involved in post-Golgi traffic really form a ring or spiral shaped polymers and sever the membrane in a GTP hydrolysis-dependent constriction? Second, if this is the case, do DRP proteins assemble directionally or randomly? As mentioned above, the current action model of DRP is mainly based on electron microscopic observation and biochemical analyses of metazoan dynamins. Recently, some high resolution microscopy techniques that overcome the diffraction barrier in existing light microscopy have become available. These include structured illumination microscopy (SIM; Gustafsson, 2005; Gustafsson et al., 2008), stimulated emission depletion microscopy (STED; Toomre and Bewersdorf, 2010), and super-resolution confocal live imaging microscopy (SCLIM; Ito et al., 2012). These techniques, when applied to fluorescent live cell imaging, have the potential to reveal the fine structure and behavior of the molecular machinery regulating organelle dynamics. They should also help to clarify the mechanisms of DRP assembly and constriction, which are the core functions of DRPs.

## Conflict of Interest Statement

The Guest Associate, Dr. Takashi Ueda, declares that, despite being affiliated to the same institution as author(s), the review process was handled objectively and no conflict of interest exists. The authors declare that the research was conducted in the absence of any commercial or financial relationships that could be construed as a potential conflict of interest.
